# Who Wants to Switch? Gauging Patient Interest in Novel Antiretroviral Therapies

**DOI:** 10.1093/ofid/ofy247

**Published:** 2018-10-22

**Authors:** Caroline B Derrick, Jan Ostermann, Sharon B Weissman, Amy Hobbie, Noor Alshareef, Andrew Weinhold, Valerie Yelverton, Nathan M Thielman

**Affiliations:** 1 Department of Medicine, School of Medicine, University of South Carolina, Columbia, South Carolina; 2 Department of Health Services Policy and Management, Arnold School of Public Health, University of South Carolina, Columbia, South Carolina; 3 Center for Health Policy and Inequalities Research, Duke Global Health Institute, Duke University, Durham, North Carolina; 4 Fachbereich Gesundheit, Pflege, Management, Hochschule Neubrandenburg, Neubrandenburg, Germany; 5 Department of Medicine, School of Medicine, Duke University, Durham, North Carolina

**Keywords:** antiretroviral therapy (ART) adherence, patient preferences, shared decision-making, treatment characteristics

## Abstract

Study participants were asked about their interest in switching to novel drug delivery systems that reduce the dosing frequency of antiretroviral regimens. Across a diverse, treatment-experienced cohort, we describe greatest interest in switching to an oral regimen taken once weekly, followed by injections taken every other month and twice-annual implants.

The past decade has witnessed a dramatic increase in the number of effective, convenient, and well-tolerated HIV combination antiretroviral therapy (cART) regimens, and novel approaches that allow for significantly decreased dosing frequency are in active development [1, 2]. Simplified regimens are widely promoted to improve adherence, and there is scientific rationale for this practice. In a meta-analysis that included 19 randomized controlled trials comparing once-daily vs twice-daily therapy, adherence (but not virologic suppression) was marginally better with once-daily vs twice-daily therapy, and lower pill burden was associated with higher adherence rates and virologic suppression [3]. Outside clinical trial settings, a large cohort study of women living with HIV identified a significant association between the use of single-tablet regimens and improvements in adherence and virologic suppression [4]. More recently, in a small single-clinic study, Hemmige et al. observed that patients on single-tablet regimens had similar adherence but better virologic suppression than those on once-daily multi-tablet regimens [5].

Barriers to access and incomplete adherence remain obstacles for effective cART and are associated with clinical and virologic failure, emergence of drug resistance, and increased risk of HIV transmission [6]. Centers for Disease Control and Prevention data indicate that, in 2015, only 81% of patients receiving care were virologically suppressed [7]. Further simplification of cART could reduce dosing-related barriers to adherence and lead to higher rates of virologic suppression. Investigational antiretroviral formulations with extended dosing intervals, including injectable or implantable extended-release agents, constitute a significant advance in antiretroviral therapy [2, 8]. Such formulations may be able to address dosing-related barriers by allowing for weekly, monthly, and potentially even longer intervals of administration. Which populations prefer these advances over current therapy options is unknown. Given the expanding number of highly effective antiretroviral regimens and newer treatment modalities, patient preferences may drive adherence. To characterize preferences for currently available and future treatment options, we describe patients’ interest in novel drug delivery systems including injectable and long-acting cARTs.

## METHODS

### Study Participants

Between February and August 2017, 263 adult persons living with HIV (PLWH) from the Infectious Diseases clinics at Duke University and the University of South Carolina were surveyed concerning their preferences for HIV treatment. Multiple recruitment methods, including flyers and invitation cards, referrals from providers, patients, and members of the community advisory board, and consecutive recruitment of patients after completion of their clinic appointments, were used to recruit samples that reflect the diversity of the treatment-experienced populations at the 2 clinics. Study activities were approved by the Institutional Review Boards of the Duke University Health System and the University of South Carolina. Written informed consent was obtained from participants at Duke University; oral informed consent was obtained from participants at the University of South Carolina.

### Survey Instrument

Trained research assistants administered in-person surveys on tablet devices at the Infectious Diseases clinics or nearby research offices. Participants were presented with the following:

Several new HIV medicines are being developed that could be taken less frequently than currently available options. Compared with your current HIV medicines, how interested would you be in switching to a new treatment that is… a single pill once a week, 2 shots in clinic every other month, or implanting and removing 2 small plastic rods about the size of matchsticks in each forearm every 6 months.

Responses were recorded on 5-point visual analog scales with 3 labels (1 = not at all interested; 3 = somewhat interested; 5 = very interested). Demographic characteristics, characteristics of patients’ current regimens, and patients’ treatment experience were documented.

### Statistical Methods

Cohort characteristics were analyzed using descriptive statistics; chi-square statistics and Student *t* tests were used to assess the significance of differences between the 2 clinic populations. Multivariate linear regression was used to jointly model patients’ interest in switching to the 3 advanced regimens as a function of sociodemographic characteristics (age, gender, education, race), HIV treatment characteristics (years on cART, most recent viral load, current side effects, long-term adverse effects), and characteristics of their current regimen (single-tablet regimen, food restrictions). The model was estimated in Stata 15 (StataCorp, College Station, TX).

## RESULTS

### Patient Characteristics

The mean age of patients surveyed was 47 years, 56% were male, and 81% were of racial or ethnic minority; of those, 95% were African American. More than half of the patient population had a high school education (42%) or less (12%). On average, patients had been on cART for 12 years; the majority of patients (59%) reported that they were currently on a single-pill once-daily regimen. Integrase inhibitor based therapies (66%) were most common, followed by non-nucleoside and protease inhibitor based regimens (24% and 20%, respectively; not shown). The majority (56%) of patients reported cART regimens with food restrictions. Most patients (82%) reported their most recent viral load to be <200 copies/mL; 22% indicated that they had missed at least 1 dose in the past 2 weeks. Current side effects were mentioned by 34% of participants; 39% reported long-term effects.

Table 1 shows patients’ interest in switching to a single pill once a week, 2 injections given in clinic every other month, and 2 small implants in the forearm every 6 months. Overall, there was greatest interest in a single weekly pill and least interest in biannual implants. In multivariate analysis, higher education was associated with greater interest in switching to a single pill once a week (*P* = .02), injections every other month (*P* < .001), and implants every 6 months (*P* = .002). Younger age (*P* = .036) and the experience of any long-term effects (*P* = .018) were associated with increased interest in switching to 2 shots every other month. Being on a 1-pill once-daily regimen was associated with lower interest in switching to a 1-pill once-weekly option (*P* = .046) and implants every 6 months (*P* = .014). Clinic site, gender, race/ethnicity, time on ART, a prior AIDS diagnosis, recent viral load, missed doses, experiencing current side effects, and current regimens with food restrictions were not associated with interest in switching to novel regimens.

**Table 1.** **Distribution and Correlates of Interest in Switching to Novel ART** **Regimens (n = 263**)

		1 Pill Once a Week	2 Shots Every Other Month	2 Implants Every 6 Months
Interest in switching, No. (%)				
Not at all interested		38 (14)	100 (38)	152 (58)
Somewhat interested		52 (20)	60 (23)	61 (23)
Very interested		173 (66)	101 (39)	5 (18)
No.		263	261	261
		β (SE)	β (SE)	β (SE)
Clinic, Duke vs South Carolina, No. (%)	132 (50.2)	0.02 (0.20)	0.22 (0.23)	0.22 (0.22)
Age, mean (SD), years	46.7 (11.8)	–0.01 (0.01)	–0.02^*^ (0.01)	–0.01 (0.01)
Gender, male vs female, No. (%)	148 (56.3)	–0.33 (0.20)	–0.12 (0.24)	0.10 (0.22)
More than high school education, yes vs no, No., (%)	109 (41.4)	0.43^*^ (0.21)	1.04^***^ (0.24)	0.72^**^ (0.23)
Race, white vs minority, No. (%)	51 (19.4)	–0.04 (0.25)	0.16 (0.30)	–0.24 (0.28)
Time on ART, mean (SD), years	12.1 (8.3)	–0.02 (0.01)	–0.03 (0.02)	–0.01 (0.01)
AIDS diagnosis, ever vs never, No. (%)	41 (15.6)	0.32 (0.25)	0.27 (0.30)	–0.12 (0.28)
Viral load <200, self-reported, yes vs no, No. (%)	215 (81.7)	0.28 (0.24)	–0.23 (0.29)	0.27 (0.27)
Missed dose, past 2 weeks, any vs none, No. (%)	58 (22.1)	–0.09 (0.23)	–0.15 (0.27)	0.00 (0.25)
Current side effects, any vs none, No. (%)	90 (34.2)	0.26 (0.20)	0.22 (0.23)	0.10 (0.22)
Long-term effects, any vs none, No. (%)	103 (39.2)	0.34 (0.20)	0.56^*^ (0.24)	0.21 (0.22)
Single-tablet regimen, yes vs no, No. (%)	155 (58.9)	–0.44^*^ (0.20)	–0.15 (0.23)	–0.44^*^ (0.22)
Food restriction, any vs none, No. (%)	148 (56.3)	0.04 (0.19)	0.27 (0.23)	0.15 (0.21)
No.	263	247	247	247

Results from a multivariate linear regression model. Dependent variables range from 1–5. Positive values for β indicate greater interest in switching. ^*^, ^**^, and ^***^ denote statistical significance at the 0.05, 0.01, and 0.001 levels, respectively. Sixteen observations were excluded from the multivariate model due to missing data on 1 or more outcome variables (n = 3) or covariates (n = 13).

Abbreviation: ART, antiretroviral therapy; No., number of patients; SD, standard deviation; SE, standard error.

## DISCUSSION

Forward-looking, patient-centered HIV care will require a greater understanding of patient preferences for emerging antiretroviral delivery systems. Which patients desire to switch, what the key motivators and perceived benefits are for switching, and ultimately which trade-offs patients are willing to make to effect switches are largely unknown. These data begin to address these issues. Across a diverse group of highly treatment-experienced PLWH, we describe greatest interest in switching to an oral regimen taken once weekly, followed by injections every other month; patients were least interested in 2 implants twice a year. It is plausible that, among the decreased-frequency dosing options presented, patients were more willing to switch to the administration modality that was most familiar, namely by pill. There was evidence of substantial preference heterogeneity; younger patients, those with higher education, those not taking a single-tablet regimen, and those who experienced at least 1 long-term effect expressed greater interest in 1 or more novel drug delivery systems.

As patients become more aware of the availability of these treatment options, formation of preferences may change, especially as the attendant risks of the novel ART delivery options are better understood. In addition, Havlir et al. describe clinic-level implementation challenges for long-acting antivirals, namely prioritization of patient populations for preferred use, clinic infrastructure requirements, and provider and patient training programs [9]. There are analogies with, and lessons may be learned from, oral contraceptive administration techniques, which require considerable patient education on delivery techniques and the critical role of clinic follow-up.

Our study has inherent limitations. First, the questions presented were part of a larger study that focused on HIV treatment preferences and shared decision-making; they were not designed to explore specific drivers of patients’ interest in switching to novel approaches. Other characteristics that may influence patients’ interest in switching to specific regimens, such as side effect profiles, long-term toxicities, and cost, could not be assessed. Second, our cohort was highly experienced and was not representative of the entire patient populations at the study sites. Although the 2 clinics in metropolitan areas in North and South Carolina are accessed by diverse patient groups, and the results did not differ between clinics, the findings may not be reflective of patient preferences in other geographic areas. More than 80% of study participants were from minority populations, and members of these populations may be particularly averse to newer medical interventions [10]. Previously, across a longitudinal cohort of PLWH in 5 southeastern states, we demonstrated that minority racial/ethnic groups were faster to discontinue cART and experience virologic failure than their counterparts, and others have described low levels of trust in HIV providers among African Americans living with HIV [11]. Our findings, interpreted in this context, call attention to the opportunity for HIV providers to build greater trust with patients by seeking to understand their preferences, communicating risks and benefits clearly, and involving them in shared antiretroviral decision-making.

Interest in longer-acting cART delivery systems varies widely among PLWH. Understanding preference heterogeneity for these novel treatment modalities may help to inform their development, predict uptake, and inform educational efforts to better engage patients in shared antiretroviral decision-making.

## Acknowledgments

***Disclaimer. ***The views expressed here do not necessarily reflect the views of the Foundation.

***Financial support. ***This work was supported by the Robert Wood Johnson Foundation (grant #73056). 

***Potential conflicts of interest. ***The authors declare that there is no conflict of interest regarding the publication of this article. All authors: no reported conflicts of interest. All authors have submitted the ICMJE Form for Disclosure of Potential Conflicts of Interest. Conflicts that the editors consider relevant to the content of the manuscript have been disclosed.
